# miRNA expression patterns in normal breast tissue and invasive breast cancers of BRCA1 and BRCA2 germ-line mutation carriers

**DOI:** 10.18632/oncotarget.5617

**Published:** 2015-09-11

**Authors:** Shoko Vos, Farhad Vesuna, Venu Raman, Paul J. van Diest, Petra van der Groep

**Affiliations:** ^1^ Department of Pathology, University Medical Center Utrecht, Utrecht, The Netherlands; ^2^ Department of Radiology, Johns Hopkins University School of Medicine, Baltimore, MD, USA

**Keywords:** breast cancer, hereditary, BRCA1, BRCA2, miRNA

## Abstract

miRNA deregulation has been found to promote carcinogenesis. Little is known about miRNA deregulation in hereditary breast tumors as no miRNA expression profiling studies have been performed in normal breast tissue of BRCA1 and BRCA2 mutation carriers. miRNA profiles of 17 BRCA1- and 9 BRCA2-associated breast carcinomas were analyzed using microarrays. Normal breast tissues from BRCA1 and BRCA2 mutation carriers (both *n* = 5) and non-mutation carriers (*n* = 10) were also included. Candidate miRNAs were validated by qRT-PCR. Breast carcinomas showed extensive miRNA alteration compared to normal breast tissues in BRCA1 and BRCA2 mutation carriers. Moreover, normal breast tissue from BRCA1 mutation carriers already showed miRNA alterations compared to non-mutation carriers. Chromosomal distribution analysis showed several hotspots containing down- or up-regulated miRNAs. Pathway analysis yielded many similarities between the BRCA1 and BRCA2 axes with miRNAs involved in cell cycle regulation, proliferation and apoptosis. Lesser known pathways were also affected, including cellular movement and protein trafficking. This study provides a comprehensive insight into the potential role of miRNA deregulation in BRCA1/2-associated breast carcinogenesis. The observed extensive miRNA deregulation is likely the result of genome-wide effects of chromosomal instability caused by impaired BRCA1 or BRCA2 function. This study's results also suggest the existence of common pathways driving breast carcinogenesis in both BRCA1 and BRCA2 germ-line mutation carriers.

## INTRODUCTION

Breast cancer, the most common cancer as well as the leading cause of death in women worldwide [1, 2], can occur both in sporadic and hereditary settings. Germ-line mutations in the BRCA1 or BRCA2 genes are the most common causes of breast cancer predisposition resulting in a 70% and 60% lifetime risk of developing breast cancer, respectively [3, 4]. These BRCA1/2-associated breast carcinomas account for 5-7% of all breast cancer cases [5]. Major progress in the classification of human breast tumors has been made by gene expression (mRNA) profiling using microarray analysis defined by luminal A, luminal B, basal-like and HER-2 subtypes [6–8].

More recently, miRNA expression profiling has been given much attention for further classification. miRNAs are small non-coding RNAs of approximately 22 nucleotides in length, that play an important role in post-transcriptional gene regulation, causing translational repression or mRNA degradation of their target mRNAs [9]. Quantitative and qualitative changes in miRNAs have been found to promote carcinogenesis, as they could lead to increased expression of oncogenes and decreased expression of tumor suppressor genes [10–18]. A global decrease in mature miRNA levels is found in tumors compared to normal tissues [19, 20], possibly attributed to the fact that many miRNAs have tumor suppressor functions. miRNA expression profiles of sporadic breast tumors show several differentially expressed miRNAs compared to normal breast tissue [21–24]. Differences in miRNA expression can partially explain breast cancer heterogeneity, such as estrogen receptor (ER) [22, 24] and progesterone receptor (PR) [22, 24] expression and presence of HER2 amplification [24]. Moreover, miRNA expression patterns can predict therapy response and resistance [25]. These findings suggest that deregulated miRNA expression is important in sporadic breast carcinogenesis. An advantage of miRNAs is that they are more resistant to degradation caused by the formalin fixation process of tissues [26]. Therefore, the opportunity to use miRNAs as biomarkers in formalin-fixed paraffin-embedded (FFPE) samples, the usual processing method applied in pathology, can be more rapidly translated to clinical practice [27]. Little is known about miRNA expression in BRCA1/2-associated breast carcinomas. The identification of target genes and pathways affected by deregulated miRNAs in BRCA1/2-associated breast carcinomas is important for attaining a better understanding of BRCA1/2-specific breast tumorigenesis and could yield new diagnostic biomarkers and therapeutic targets. The aims of this study were: (1) to analyze differences in miRNA expression profiles between BRCA1/2*-*associated breast carcinomas, normal breast tissue from BRCA1 and BRCA2 germ-line mutation carriers, and normal breast tissue from non-mutation carriers; (2) to obtain more insight into BRCA1/2*-*associated carcinogenesis by identification of target genes and pathways regulated by miRNAs.

## RESULTS

### Clinicopathologic characteristics of tissue samples

The patient samples (all FFPE) consisted of 5 classes: BRCA1-associated breast carcinomas (BRCA1-C) (*n* = 17); BRCA*2*-associated breast carcinomas (BRCA2-C) (*n* = 9); normal breast tissue from BRCA1 germ-line mutation carriers (BRCA1-N) (*n* = 5) and BRCA2 germ-line mutation carriers (BRCA2-N) (*n* = 5), both derived from prophylactic mastectomies; and normal breast tissue from non-mutation carriers derived from mammoplasty specimens (healthy-N) (*n* = 10). For external validation of specific miRNAs by qRT-PCR, a second, independent, cohort of patient samples was used. This cohort consisted of a total of 60 FFPE samples, obtained from the same archives. The patient samples also consisted of 5 classes: BRCA1-C (*n* = 15); BRCA2-C (*n* = 15); BRCA1-N (*n* = 10); BRCA2-N (*n* = 10); and Healthy-N (*n* = 10). Patient characteristics are shown in Tables 1a and 1b for the first and second cohorts, respectively. Details on the characterization of patient samples are given in the Supplementary methods. Average age at diagnosis in the BRCA1-associated breast carcinomas was 46.1 years (range 21 - 81) in the first cohort, and 41.3 years (range 28 - 56) in the second cohort. The tumors were mainly of ductal type (58.8% and 93.3% in the first and second cohorts, respectively), and ER, PR, and HER2 negative (58.8%, 76.5% and 82.4%, respectively in the first cohort; and 80%, 86.7% and 80%, respectively in the second cohort). The patients with BRCA2-associated breast cancer had an average age at diagnosis of 46.7 years (range 21 - 66) in the first cohort, and 45.8 years (range 27 - 67) in the second cohort. These tumors were also mainly of ductal type (88.9% and 100% in the first and second cohorts, respectively), ER positive (55.6% and 80% in the first and second cohorts, respectively), PR negative (66.7%) in the first cohort and more PR positive (53.3%) in the second cohort, and HER2 negative (100%) in the first cohort and more HER2 positive (46.7%) in the second cohort. The average age of patients of whom normal breast tissues were used, was 33.6, 36.2, and 30.4 years for BRCA1-N, BRCA2-N, and Healthy-N in the first cohort, respectively; and 35.5, 41.0, and 40.4 years in the second cohort, respectively.

**Table 1a:** Patient characteristics, initial cohort

Characteristics	n (%)				
	BRCA1-cancers	BRCA2 cancers	BRCA1 normal tissue	BRCA2 normal tissue	Healthy normal tissue
Age in years (mean ± SD)	46.1 ± 16.4 (range 21 - 81)	46.7 ± 14.8 (range 21 - 66)	33.6 ± 8.4 (range 26 - 49)	36.2 ± 7.7 (range 27 - 49)	30.4 ± 6.4 (range 21 - 38)
Grade					
I	1 (5.9)	0 (0)	NA	NA	NA
II	5 (29.4)	4 (44.4)	NA	NA	NA
III	11 (64.7)	5 (55.6)	NA	NA	NA
Tumor type					
Ductal	10 (58.8)	8 (88.9)	NA	NA	NA
Lobular	3 (17.6)	1 (11.1)	NA	NA	NA
Medullary	3 (17.6)	0 (0)	NA	NA	NA
Metaplastic	1 (5.9)	0 (0)	NA	NA	NA
Estrogen receptor					
Negative	10 (58.8)	4 (44.4)	NA	NA	NA
Positive	7 (41.2)	5 (55.6)	NA	NA	NA
Progesterone receptor					
Negative	13 (76.5)	6 (66.7)	NA	NA	NA
Positive	4 (23.5)	3 (33.3)	NA	NA	NA
HER2 status					
Negative	14 (82.4)	9 (100)	NA	NA	NA
Positive	3 (17.6)	0 (0)	NA	NA	NA

NA = not applicable.

**Table 1b:** Patient characteristics, second cohort

Characteristics	n (%)				
	BRCA1 cancers	BRCA2 cancers	BRCA1 normal tissue	BRCA2 normal tissue	Healthy normal tissue
Age in years (mean ± SD)	41.3 ± 9.1 (range 28 - 56)	45.8 ± 10.1 (range 27 - 67)	35.5 ± 6.5 (range 26 - 40)	41.0 ± 18.4 (range 28 - 54)	40.4 ± 12.5 (range 21 - 60)
Grade					
I	0 (0)	0 (0)	NA	NA	NA
II	1 (6.7)	3 (20)	NA	NA	NA
III	13 (86.7)	12 (80)	NA	NA	NA
Missing	1 (6.7)	0 (0)	NA	NA	NA
Tumor type					
Ductal	14 (93.3)	15 (100)	NA	NA	NA
Missing	1 (6.7)	0 (0)	NA	NA	NA
Estrogen receptor					
Negative	12 (80)	3 (20)	NA	NA	NA
Positive	3 (20)	12 (80)	NA	NA	NA
Progesterone receptor					
Negative	13 (86.7)	7 (46.7)	NA	NA	NA
Positive	2 (13.3)	8 (53.3)	NA	NA	NA
HER2 status					
Negative	12 (80)	7 (46.7)	NA	NA	NA
Positive	2 (13.3)	7 (46.7)	NA	NA	NA
Missing	1 (6.7)	1 (6.7)	NA	NA	NA

NA = not applicable.

### miRNA microarray analysis

Of the 2006 human miRNAs on the microarray, 862 miRNAs remained after filtering on low expression variation. Differential expression analysis between the five classes (BRCA1-C, BRCA2-C, BRCA1-N, BRCA2-N, and Healthy-N) was performed. The numbers of differentially expressed miRNAs with fold change (FC) ≥ │1.5│and statistical significance (False Discovery Rate (FDR) < 0.05) for each comparison are shown in Table 2. The BRCA2-C *vs*. Healthy-N comparison yielded many more differentially expressed miRNAs (*n* = 600) compared to the BRCA1-C *vs*. Healthy-N comparison (*n* = 269). Moreover, the BRCA2-C *vs*. Healthy-N comparison yielded mainly up-regulated miRNAs in contrast to down-regulated miRNAs in the BRCA1-C *vs*. Healthy-N comparison. 317 miRNAs were differentially expressed in the BRCA1-C *vs*. BRCA2-C comparison. There were 150 differentially expressed miRNAs between BRCA1-N and Healthy-N. However, the BRCA2-N *vs*. Healthy-N comparison yielded no significant results. Potential correlations between biological differences (irrespective of BRCA status) and miRNA expression, which could have influenced above mentioned results, were also investigated. miRNA expression did not show any significant correlations with age (≤50 *vs*. >50 years), grade (2 *vs*. 3), the presence of lymph node metastases, or expression of PR, ER, CK5/6, CK14, EGFR, or Ki-67 (<20 *vs*. ≥20%), irrespective of BRCA status (data not shown). However, HER2 expression and tumor type were associated with differences in miRNA expression, irrespective of BRCA status. 85 miRNAs were significantly differentially expressed between HER2 positive and negative tumors (see Supplementary table SI). One miRNA (miR-4633-5p) was significantly differentially expressed between ductal and lobular breast carcinomas (FC = 1.71, FDR = 0.0318, data not shown).

**Table 2:** Number of differentially expressed miRNAs between classes

Class	Class compared to	Total differentially expressed miRNAs	Up-regulated miRNAs	Down-regulated miRNAs
BRCA1 cancers	Healthy normal tissue	269	90	179
BRCA1 cancers	BRCA1 normal tissue	145	41	104
BRCA1 normal tissue	Healthy normal tissue	150	55	95
BRCA2 cancers	Healthy normal tissue	600	360	240
BRCA2 cancers	BRCA2 normal tissue	96	8	88
BRCA2 normal tissue	Healthy normal tissue	0	0	0
BRCA1 cancers	BRCA2 cancers	317	121	196
BRCA1 normal tissue	BRCA2 normal tissue	0	0	0

miRNAs with with fold change ≥ |1.5| and statistical significance (False Discovery Rate < 0.05) are included in this analysis using unpaired *t*-test for unequal variance.

We focused on BRCA1-C *vs*. BRCA1-N and BRCA2-C *vs*. BRCA2-N comparisons, as differentially expressed miRNAs from these comparisons could play a role in BRCA1/2-associated breast carcinogenesis. The BRCA1-C *vs*. BRCA1-N comparison yielded 145 miRNAs compared to 96 in the BRCA2-C *vs*. BRCA2-N comparison. These comparisons had 53 miRNAs in common. The shared miRNAs, and the BRCA1 and BRCA2 axis-specific miRNAs were ranked based upon FDR and subsequently on fold change, and the top 10 miRNAs are shown in Tables 3, 4, 5. Of these, the following miRNAs were selected for qRT-PCR validation, based on assay availability: miR-99a, miR-210, miR-21, miR-183, miR-378, miR-153, miR-4443, miR-1287, let-7b and miR-551b.

**Table 3:** Top 10 differentially expressed miRNAs shared between the BRCA1 and BRCA2 axis

miRNA	Chromosomal location	Cancer vs. normal in BRCA1 carriers			Cancer vs. normal in BRCA2 carriers		
		Unadjusted p-value	FDR	FC	Unadjusted p-value	FDR	FC
**Up-regulated**							
hsa-miR-3676-5p	Chr17: 8090493-8090577 [+]	0.0034	0.0212	1.67	0.0000	0.0212	2.47
hsa-miR-937-5p	Chr8q24.3: 144895127-144895212 [−]	0.0052	0.0286	1.55	0.0046	0.0286	1.64
**Down-regulated**							
hsa-miR-99a-3p	Chr21q21.1: 17911409-17911489 [+]	0.0000	0.0000	25.29	0.0000	0.0048	25.79
hsa-miR-204-5p	Chr9q21.12: 73424891-73425000 [−]	0.0000	0.0000	67.98	0.0000	0.0048	227.67
hsa-miR-4328	ChrX: 78156691-78156746 [−]	0.0000	0.0000	17.93	0.0001	0.0048	33.96
hsa-miR-136-3p	Chr14q32.2: 101351039-101351120 [+]	0.0000	0.0000	58.28	0.0001	0.0048	67.82
hsa-miR-99a-5p	Chr21q21.1: 17911409-17911489 [+]	0.0000	0.0000	6.76	0.0000	0.0048	13.60
hsa-miR-125b-5p	Chr11q24.1: 121970465-121970552 [−]	0.0000	0.0000	5.35	0.0001	0.0058	11.97
hsa-miR-100-5p	Chr11q24.1: 122022937-122023016 [−]	0.0000	0.0001	4.27	0.0001	0.0082	10.01
hsa-miR-4770	ChrX: 6301947-6302004 [−]	0.0000	0.0001	14.09	0.0000	0.0031	35.44
hsa-miR-195-5p	Chr17p13.1: 6920934-6921020 [−]	0.0000	0.0001	4.65	0.0001	0.0053	10.82
hsa-miR-199b-5p	Chr9q34.11: 131007000-131007109 [−]	0.0000	0.0001	5.77	0.0006	0.0195	10.57

Ranking of miRNAs based upon FDR (smallest to largest) and FC (largest to smallest). Chromosomal locations are based upon NCBI Gene results and GRCh37.p5 coordinates. miRNAs marked in yellow are selected for qRT-PCR validation. This selection is based upon above described ranking and availability of qRT-PCR primers. FDR = false discovery rate. FC = fold change.

**Table 4:** Top 10 differentially expressed miRNAs specifically altered between normal tissues and cancers of BRCA1 carriers

miRNA	Chromosomal location	Unadjusted p-value	FDR	FC
**Up-regulated**				
hsa-miR-1307-3p	Chr10: 105154010-105154158 [−]	0.0000	0.0001	1.70
hsa-miR-210	Chr11p15.5: 568089-568198 [−]	0.0000	0.0002	4.61
hsa-miR-3162-3p	Chr11: 59362550-59362631 [−]	0.0000	0.0002	2.41
hsa-miR-155-5p	Chr21q21.3: 26946292-26946356 [+]	0.0000	0.0003	3.59
hsa-miR-21-5p	Chr17q23.1: 57918627-57918698 [+]	0.0000	0.0004	3.62
hsa-miR-4306	Chr13: 100295313-100295403 [+]	0.0000	0.0004	1.83
hsa-miR-183-5p	Chr7q32.2: 129414745-129414854 [−]	0.0000	0.0007	7.19
hsa-miR-185-5p	Chr22q11.21: 20020662-20020743 [+]	0.0000	0.0009	2.47
hsa-miR-574-5p	Chr4: 38869653-38869748 [+]	0.0000	0.0009	2.71
hsa-miR-4455	Chr4: 185859537-185859594 [−]	0.0001	0.0012	3.27
**Down-regulated**				
hsa-miR-378a-5p	Chr5q32: 149112388-149112453 [+]	0.0000	0.0000	29.07
hsa-miR-153	Chr2q35: 220158833-220158922 [−]	0.0000	0.0000	18.90
hsa-miR-29a-5p	Chr7q32.3: 130561506-130561569 [−]	0.0000	0.0004	7.32
hsa-miR-1258	Chr2q31.3: 180725563-180725635 [−]	0.0000	0.0004	12.82
hsa-miR-335-3p	Chr7q32.2: 130135952-130136045 [+]	0.0000	0.0006	14.98
hsa-miR-6500-3p	Chr1: 51525690-51525775 [+]	0.0000	0.0006	10.69
hsa-let-7i-3p	Chr12q14.1: 62997466-62997549 [+]	0.0000	0.0007	47.60
hsa-miR-411-5p	Chr14q32.31: 101489662-101489757 [+]	0.0000	0.0007	47.60
hsa-miR-219-5p	Chr6p21.32: 33175612-33175721 [+]	0.0000	0.0009	15.36
hsa-miR-139-5p	Chr11q13.4: 72326107-72326174 [−]	0.0000	0.0009	11.10

Ranking of miRNAs based upon FDR (smallest to largest) and FC (largest to smallest). Chromosomal locations are based upon NCBI Gene results and GRCh37.p5 coordinates. miRNAs marked in yellow are selected for qRT-PCR validation. This selection is based upon above described ranking and availability of qRT-PCR primers. FDR = false discovery rate. FC = fold change.

**Table 5:** Top 10 differentially expressed miRNAs specifically altered between normal tissues and cancers of BRCA2 carriers

miRNA	Chromosomal location	Unadjusted p-value	FDR	FC
**Up-regulated**				
hsa-miR-4778-5p	Chr2: 66585381-66585460 [−]	0.0002	0.0105	1.62
hsa-miR-4443	Chr3: 48238054-48238106 [+]	0.0005	0.0169	2.05
hsa-miR-5010-5p	Chr17: 40666206-40666325 [+]	0.0022	0.0333	1.62
hsa-miR-1287	Chr10q24.2: 100154975-100155064 [−]	0.0039	0.0434	1.91
hsa-miR-663b	Chr2: 133014539-133014653 [−]	0.0045	0.0459	2.05
hsa-miR-4688	Chr11: 46397952-46398034 [+]	0.0060	0.0489	1.61
**Down-regulated**				
hsa-miR-664b-5p	ChrX: 153996871-153996931 [+]	0.0000	0.0048	2.72
hsa-let-7b-5p	Chr22q13.31: 46509566-46509648 [+]	0.0005	0.0169	4.37
hsa-miR-29b-1-5p	Chr7q32.3: 130562218-130562298 [−]	0.0007	0.0195	17.92
hsa-miR-551b-3p	Chr3q26.2: 168269642-168269737 [+]	0.0009	0.0242	63.99
hsa-let-7g-5p	Chr3p21.1: 52302294-52302377 [−]	0.0010	0.0255	4.21
hsa-miR-650	Chr22q11.22: 23165270-23165365 [+]	0.0011	0.0264	1.97
hsa-miR-29a-3p	Chr7q32.3: 130561506-130561569 [−]	0.0012	0.0268	3.81
hsa-miR-1234-3p	Chr8: 145625476-145625559 [−]	0.0012	0.0268	1.86
hsa-miR-224-3p	ChrXq28: 151127050-151127130 [−]	0.0016	0.0295	45.54
hsa-miR-148a-3p	Chr7p15.2: 25989539-25989606 [−]	0.0017	0.0295	4.97

Ranking of miRNAs based upon FDR (smallest to largest) and FC (largest to smallest). Chromosomal locations are based upon NCBI Gene results and GRCh37.p5 coordinates. miRNAs marked in yellow are selected for qRT-PCR validation. This selection is based upon above described ranking and availability of qRT-PCR primers. FDR = false discovery rate. FC = fold change.

### miRNA validation by qRT-PCR

miRNA validation by qRT-PCR was performed in the same samples as used for microarray analysis together with an independent cohort of samples, yielding a combination of internal and external validation of the initial miRNA microarray results. By doing this, more well founded results would be obtained that are also generalizable to other cases. All miRNAs selected for qRT-PCR validation, except miR-1287, were differentially expressed in the same direction in qRT-PCR as in microarray analysis between the invasive breast carcinomas and the asymptomatic normal breast tissues of BRCA1 and BRCA2 germ-line mutation carriers (see Table 6). However, qRT-PCR analysis appeared to be more sensitive compared to microarray analysis. miRNAs miR-210, miR-21, miR-183 and miR-153 were specifically differentially expressed between the BRCA1-C and BRCA1-N stages in microarray analysis and were confirmed by qRT-PCR analysis. Only miR-378 was not significantly differentially expressed between the BRCA1-C and BRCA1-N stages in qRT-PCR analysis. Noteworthy, all the microarray specifically differentially miRNAs between BRCA1-C and BRCA1-N were also significantly differentially expressed between the BRCA2-C and BRCA2-N stages in qRT-PCR analysis.

**Table 6:** miRNA validation by qRT-PCR: test statistics Invasive breast carcinomas *vs*. asymptomatic normal breast tissues of BRCA1 and BRCA2 germ-line mutation carriers

	Let-7b	miR-153	miR-183	miR-210	miR-378	miR-4443	miR-551b	miR-1287	miR-21	miR-99a
**Kruskal-Wallis test**	31.904	26.434	76.684	74.067	26.808	23.231	31.321	5.655	77.210	63.637
**Signifance level**	0.000	0.000	0.000	0.000	0.000	0.000	0.000	0.226	0.000	0.000

Of miRNAs specifically differentially expressed between the BRCA2-C and BRCA2-N stages in microarray analysis (let-7b, miR-4443, miR-551b, miR-1287), miR-4443 and miR-1287 were not significantly differentially expressed between these two classes in qRT-PCR analysis. Let-7b was found to be specifically deregulated between invasive breast carcinomas and asymptomatic normal breast tissues in BRCA2 germ-line mutation carriers. miR-551b was found to be significantly differentially expressed between the BRCA2-C and BRCA2-N stages as well as between the BRCA1-C *vs*. BRCA1-N stages. miR-4443 was however found to be significantly differentially expressed between the BRCA1-C and BRCA1-N stages by qRT-PCR. qRT-PCR analysis of miR-99a confirmed the microarray results showing that it was down-regulated in both BRCA1- and BRCA2-associated breast carcinomas compared to their normal breast tissue counterparts.

### Chromosomal distribution

**Figure 1:**
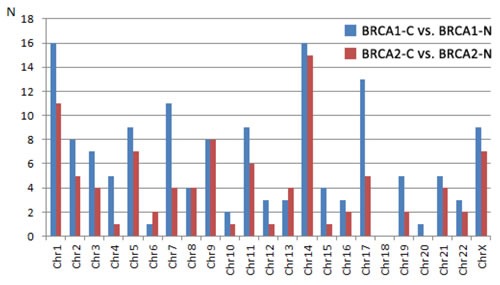
Number of differentially expressed miRNAs per chromosome from the comparison between cancers and normal tissue from BRCA1 and BRCA2 carriers, respectively Explanation: Fisher's exact test: 10.200, p-value 0.989. BRCA1-C = BRCA1-associated breast carcinomas; BRCA2-C = BRCA2-associated breast carcinomas; BRCA1-N = normal breast tissue from BRCA1 germ-line mutation carriers; BRCA2-N = normal breast tissue from BRCA2 germ-line mutation carriers.

**Figure 2:**
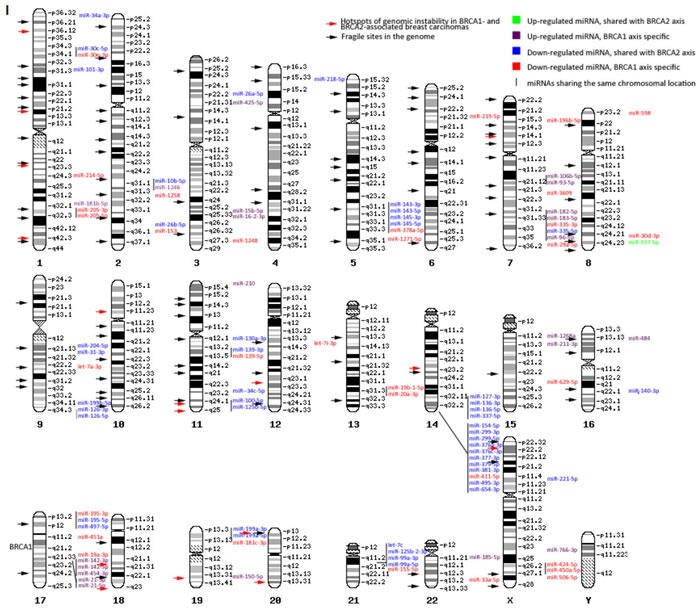

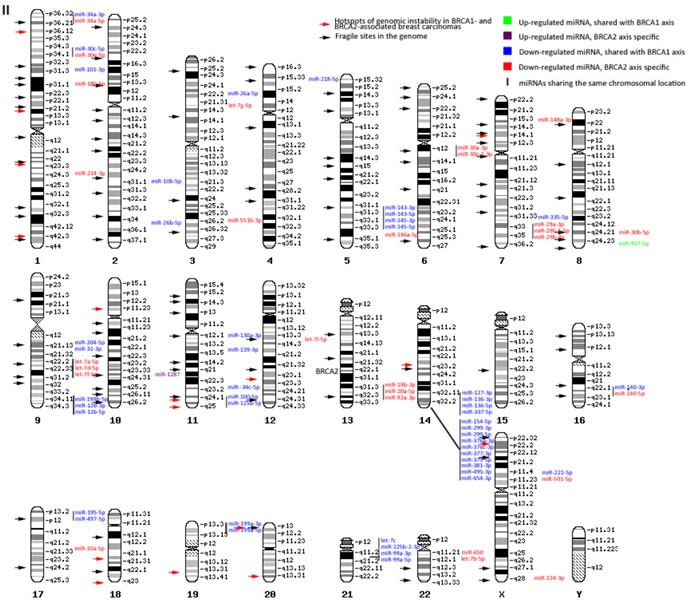
Chromosomal location of differentially expressed miRNAs between the normal tissue and cancers of BRCA1 and BRCA2 carriers, respectively I. Chromosomal distribution of miRNAs differentially expressed between normal tissue and cancers of BRCA1 carriers. II. Chromosomal distribution of miRNAs differentially expressed between normal tissue and cancers of BRCA2 carriers. All differentially expressed miRNAs from both comparisons with fold change ≥ │1.5│and false discovery rate < 0.05 and known exact chromosomal location are presented. Within chromosomes a mixture of up- and down-regulated miRNAs can be seen. Several hotspots (≥ 4 miRNAs at the same locus) can be seen, in which the miRNAs show a similar direction of deregulation. The miRNA locations partly overlap with known hotspots of chromosomal instability in BRCA1- and BRCA2-associated carcinomas and fragile sites in the genome, in which miRNAs are often located.

Chromosomal distribution of the differentially expressed miRNAs from the BRCA1-C *vs*. BRCA1-N (*n* = 145) and BRCA2-C *vs*. BRCA2-N (*n* = 96) comparisons were also investigated (Figure 1). Chromosomes 4, 7, 10, 12, 17, and 19 showed a higher number of deregulated miRNAs in the BRCA1 axis, while chromosomes 6 and 13 showed a higher number of miRNAs deregulated in the BRCA2 axis. However, the chromosomal distribution between the two axes was not significantly different (Fischer's exact test: *p* = 0.989). A more detailed view is given in Figure 2, showing the differentially expressed miRNAs at their exact localization on the chromosomes, their direction of change, and whether these miRNAs are shared between the BRCA1 and BRCA2 axis. Only miRNAs of which the exact localization within the chromosomes was known are included in this figure. The amount of miRNAs of which the localization was not known was *n* = 44 for the BRCA1-C *vs*. BRCA1-N comparison and *n* = 20 for the BRCA2-C *vs*. BRCA2-N comparison). Within chromosomes, a mixture of up- and down-regulated miRNAs was seen, although within hotspots (≥ 4 miRNAs at the same locus) the miRNAs showed a similar direction of deregulation. Shared hotspots between the BRCA1 and BRCA2 axes were 5q32, 14q32.2, 14q32.31, and 21q21.1 (all down-regulated). A BRCA1-specific hotspot was 7q32.2 (mainly up-regulated miRNAs). The chromosomal location of 3 deregulated miRNAs (miR-100-5p, miR-125b-5p, and miR-150-5p) matches hotspot regions of genomic instability in BRCA1/2-associated breast carcinomas [28]. Several more miRNAs are located at fragile sites in the genome [29], 20.8% and 22.4% for the BRCA1 and BRCA2 axis, respectively (Figure 2).

### Unsupervised clustering

**Figure 3:**
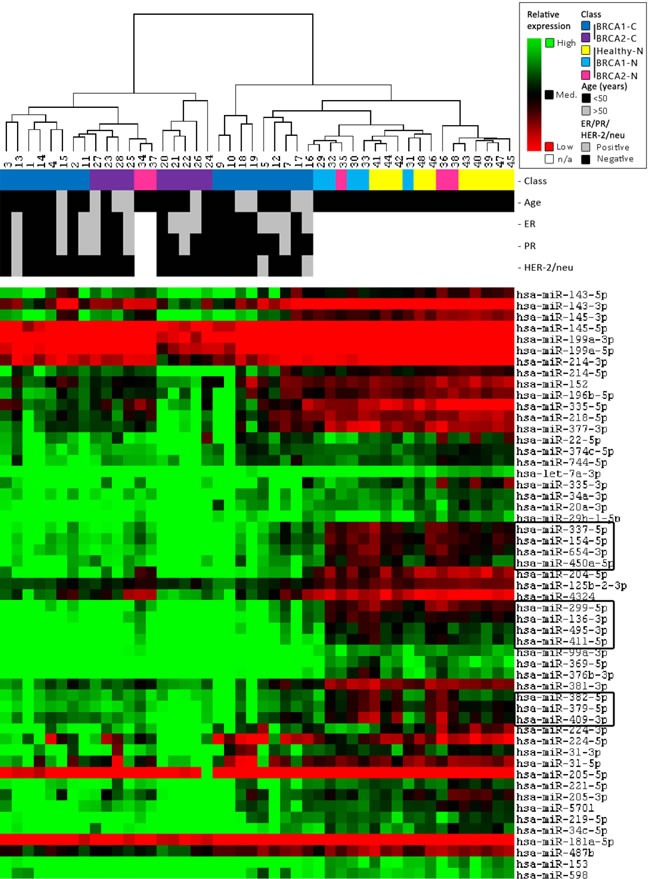

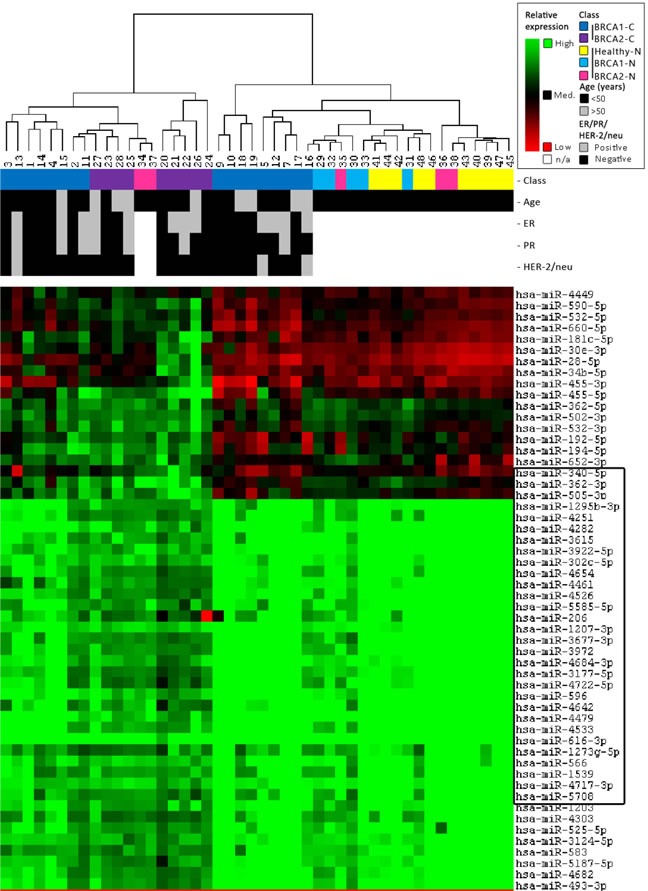
Unsupervised clustering results Clustering was performed on both all samples and all miRNAs using a Self-Organizing Map algorithm. From the total heatmap, the most distinguishing parts between the classes are shown in this figure. For further information on the figure, see the legend in the top right corner. The clustering indicates that miRNAs can separate carcinomas (BRCA2 > BRCA1) from the normal breast tissue of both BRCA1/2 and non-mutation carriers. In general, normal breast tissue of BRCA1/2-mutation carriers clusters more with normal breast tissue from non-carriers. However, some of them cluster better with BRCA1/2-associated breast carcinomas.

Unsupervised clustering analysis of miRNA microarray results yielded seven individual clusters in two main groups, largely separating breast cancers (BRCA2-C more than BRCA1-C) from the BRCA1/2-N and Healthy-N normal breast tissue samples. The most distinguishing parts between the classes of the full heatmap are shown in Figure 3. In general, most BRCA1/2-N tissues clustered with healthy-N tissue. However, some of them clustered with BRCA1/2-C. miRNAs showing a significantly different expression pattern between these two groups are surrounded with a box (Figure 3). These miRNAs, which might distinguish BRCA1 and BRCA2 germ-line carriers with a higher risk from those with a lower risk of developing breast cancer, are involved in pathways such as integrin signaling, estrogen receptor signaling, breast cancer regulation by Stathmin1, HIF1α Signaling, Wnt/β-catenin Signaling, and p53 signaling (IPA analysis). Patient samples similar in age, ER, PR, and HER2 status did not cluster together.

### Target gene and pathway analysis by DIANA-mirPath

Pathway analysis of differentially expressed miRNAs between the BRCA1-C and BRCA1-N with fold change ≥ │2.0│ and FDR < 0.05 yielded 216 significantly enriched pathways. These miRNAs target multiple genes in pathways involved in cell proliferation, apoptosis, protein ubiquitination, gene transcription, and extra-cellular matrix (ECM-) receptor signaling. There were 208 significantly enriched pathways for the BRCA2-C *vs*. BRCA2-N comparison, showing many pathways specific for the BRCA1 or BRCA2 axis (data not shown). The top 10 enriched pathways and a number of biologically interesting pathways (*p* < 0.05) are shown in Tables 7–8. A more detailed overview containing the gene names and miRNA entities for each enriched pathway is given in Supplementary table SII. Specific target analysis for the miRNAs selected for qRT-PCR was performed using IPA (*p* < 0.05) (Tables 9–10). The number of targeted genes varied from 0 (hsa-miR-99a-3p) to 19 (hsa-miR-21-5p and hsa-miR-4443).

**Table 7:** DIANA-mirPath pathway enrichment analysis for miRNAs differentially expressed between normal tissues and cancers of BRCA1 carriers

Rank	KEGG pathway	*p*-value	N of genes	N of miRNAs
1	Prion diseases	0.0000	3	3
2	Pathways in cancer	0.0000	154	20
3	PI3K-Akt signaling pathway	0.0000	175	21
4	Prostate cancer	0.0000	58	23
5	Wnt signaling pathway	0.0000	100	31
6	Neurotrophin signaling pathway	0.0000	70	21
7	Axon guidance	0.0000	69	18
8	MAPK signaling pathway	0.0000	120	18
9	TGF-beta signaling pathway	0.0000	47	16
10	Ubiquitin mediated proteolysis	0.0000	76	13
15	ErbB signaling pathway	0.0000	52	15
17	Transcriptional misregulation in cancer	0.0000	78	15
26	ECM-receptor interaction	0.0000	22	8
32	p53 signaling pathway	0.0000	37	12

Table showing significantly enriched pathways (*p* < 0.05) and the number of genes targeted by how many of the top 71 miRNAs differentially expressed between BRCA1-C and BRCA1-N conditions with fold change ≥ |2.0| and FDR < 0.05 using DIANA-mirPath pathway enrichment analysis. The ranking is based upon a combination of *p*-value, N of genes and N of miRNAs.

**Table 8:** DIANA-mirPath pathway enrichment analysis for miRNAs differentially expressed between normal tissues and cancers of BRCA2 carriers

Rank	KEGG pathway	*p*-value	N of genes	N of miRNAs
1	Prion diseases	0.0000	1	4
2	Protein digestion and absorption	0.0000	33	13
3	Amoebiasis	0.0000	44	15
4	Axon guidance	0.0000	70	17
5	ECM-receptor interaction	0.0000	31	17
6	Small cell lung cancer	0.0000	46	17
7	Long-term potentiation	0.0000	43	18
8	ErbB signaling pathway	0.0000	53	19
9	Ubiquitin mediated proteolysis	0.0000	76	19
10	Insulin signaling pathway	0.0000	70	19
14	MAPK signaling pathway	0.0000	126	25
15	Pathways in cancer	0.0000	176	26
19	PI3K-Akt signaling pathway	0.0000	197	36
20	Wnt signaling pathway	0.0000	101	38
22	TGF-beta signaling pathway	0.0000	49	18
27	p53 signaling pathway	0.0000	40	19
31	Transcriptional misregulation in cancer	0.0000	76	20
53	HIF-1 signaling pathway	0.0010	46	10

**Table 9:** IPA target gene analysis on miRNAs specifically altered between normal tissue and cancers of BRCA1 carriers and selected for qRT-PCR

miRNA	Targeted genes	Associated pathways
hsa-miR-153	ACVR1B	Epithelial adherens junction, PPARα/RXRα activation, TGF-β signaling, Wnt/β-catenin signaling
	BCL2	Apoptosis, glucocorticoid receptor signaling, p53 signaling, PEDF signaling, PI3K/AKT signaling, PTEN signaling, TGF-β signaling, VEGF signaling
	CBX5	ATM signaling
	DNM3	Clathrin-mediated endocytosis signaling, remodeling of epithelial adherens junctions
	DVL3	Embryonic stem cell pluripotency, regulation of the epithelial-mesenchymal transition pathway, Wnt/β-catenin signaling
	FOXO3	Glucocorticoid receptor signaling, IGF-1 signaling, insulin receptor signaling, PI3K/AKT signaling, PTEN signaling, PXR/RXR activation, VEGF signaling
	RAB7A	Clathrin-mediated endocytosis signaling, remodeling of epithelial adherens junctions
	SNAI1	Epithelial adherens junction signaling, ILK signaling, regulation of the epithelial-mesenchymal transition pathway
hsa-miR-183-5p	BTRC	Cyclins and cell cycle regulation, NF-κB signaling, Wnt/β-catenin signaling
	FGF9	Actin cytoskeleton signaling, clathrin-mediated endocytosis signaling, FGF signaling, regulation of the epithelial-mesenchymal transition pathway
	FOXO1	ErbB signaling, IGF-1 signaling, PI3K/AKT signaling, PTEN signaling, VEGF signaling
	PRKACB	Breast cancer regulation by Stathmin1, CDK5 signaling, NF-κB signaling PPARα/RXRα activation, tight junction signaling, cAMP-mediated signaling, eNOS signaling
	RAD50	ATM signaling, DNA double-strand break repair by homologous recombination, DNA double-strand break repair by non-homologous end joining, hereditary breast cancer signaling, role of BRCA1 in DNA damage response, role of CHK proteins in cell cycle checkpoint control, telomere extension by telomerase
hsa-miR-21-5p	ACTA2	Actin cytoskeleton signaling, gap junction/integrin/tight junction signaling, VEGF signaling
	BMPR2	BMP signaling, adherens junction signaling, PPARα/RXRα activation, TGF-β signaling
	BTG2	Cell cycle regulation
	CDK6	Cyclins and cell cycle regulation, HER-2 signaling, hereditary breast cancer signaling
	CDKN1A	See CDK6, role of BRCA1 in DNA damage response, p53 signaling, PI3K/AKT signaling, PTEN signaling
	CFL2	Actin cytoskeleton signaling, role of tissue factor in cancer
	E2F1	Breast cancer regulation by Stathmin1, cyclins and cell cycle regulation, hereditary breast cancer signaling, p53 signaling, role of BRCA1 in DNA damage response
	FAS(LG)	Apoptosis, p38 MAPK signaling, p53 signaling, PEDF signaling, PTEN signaling
	FGF1	Actin cytoskeleton signaling, clathrin-mediated endocytosis signaling, epithelial adherens junction signaling, FGF signaling, regulation of the epithelial-mesenchymal transition pathway
	JAG1	Notch signaling, regulation of EMT pathway
	PIK3R1	Actin cytoskeleton signaling, breast cancer regulation by Stathmin1, HER-2 signaling in breast cancer, hereditary breast cancer signaling,p53 signaling, PTEN signaling, regulation of EMT pathway, VEGF signaling
	PTEN	Adherens junction signaling, ErbB signaling, hereditary breast cancer signaling, integrin signaling, p53 signaling, PI3K/AKT signaling, PTEN signaling, role of tissue factor in cancer, tight junction signaling
	SERPINB5	p53 signaling
	TGFBR2	Adherens junction signaling, glucocorticoid receptor signaling, p38 MAPK signaling, PPARα/RXRα activation, TGF-β signaling, tight junction signaling, Wnt/β-catenin signaling
	TNF	Apoptosis, glucocorticoid receptor signaling, p38 MAPK signaling, PPAR signaling, tight junction signaling
hsa-miR-210	ACVR1B	Adherens junction signaling, PPARα/RXRα activation, TGF-β signaling, Wnt/β-catenin signaling
	E2F3	Breast cancer regulation by Stathmin1, cyclins and cell cycle regulation, estrogen-mediated S-phase entry, role of BRCA1 in DNA damage response
	FGFRL1	FGF signaling, NF-κB signaling, PTEN signaling, regulation of EMT pathway
	PTPN1	Caveolar-mediated endocytosis signaling, insulin receptor signaling, JAK/Stat signaling, protein kinase A signaling
hsa-miR-378a-5p	SUFU	Molecular mechanisms of cancer

All *p* < 0.05, using IPA.

**Table 10:** IPA target gene analysis on miRNAs specifically altered between normal tissue and cancers of BRCA2 carriers and selected for qRT-PCR

miRNA	Targeted genes	Associated pathways
hsa-let-7b-5p	ACTA1	ILK signaling, integrin signaling, regulation of actin-based motility by Rho, remodeling of epithelial adherens junctions, tight junction signaling, VEGF Signaling
	ACVR1B	Epithelial adherens junction signaling, PPARα/RXRα activation, TGF-β signaling, Wnt/β-catenin signaling
	BTG2	Cell cycle regulation
	CDK6	Cyclins and cell cycle regulation, HER-2 signaling, hereditary breast cancer signaling
	DVL3	Embryonic stem cell pluripotency, regulation of the epithelial-mesenchymal transition pathway, Wnt/β-catenin signaling
	E2F2	Breast cancer regulation by Stathmin1, cyclins and cell cycle regulation, estrogen-mediated S-phase entry, role of BRCA1 in DNA damage response, role of CHK proteins in cell cycle checkpoint control
	FAS(LG)	Apoptosis, p38 MAPK signaling, p53 signaling, PEDF signaling, PTEN signaling
	FGF11	Actin cytoskeleton signaling, clathrin-mediated endocytosis signaling, FGF signaling regulation of the epithelial-mesenchymal transition pathway
	PPP2R2A	Breast cancer regulation by Stathmin1, cyclins and cell cycle regulation, ERK/MAPK signaling, ILK signaling, telomerase signaling, tight junction signaling, Wnt/β-catenin signaling
hsa-miR-1287	ERBB3	ErbB signaling
	HTR2B	G-protein coupled receptor signaling, gap junction signaling
	RBL2	G1/S checkpoint regulation, role of BRCA1 in DNA damage response
	RPS20	EIF2 signaling, mTOR signaling
hsa-miR-4443	CDH5	Wnt/β-catenin signaling
	CLDN18	Tight junction signaling
	DLL4	Notch signaling
	F2RL2	Tight junction signaling
	FAS	Apoptosis, p38 MAPK signaling, p53 signaling, PEDF signaling
	IL1RN	NF-κB signaling, p38 MAPK signaling, PPAR signaling
	ITGA2B	Caveolar-mediated endocytosis signaling, integrin signaling
	LRP6	Wnt/β-catenin signaling
	NCOA1	Androgen signaling, estrogen receptor signaling, HIF1α signaling, PPAR signaling
	NTRK3	NF-κB signaling, PTEN signaling
	PCK1	Estrogen receptor signaling
	PLCL1	Gap junction signaling, PPARα/RXRα activation, protein kinase A signaling
	PRKAA2	AMPK signaling, eNOS signaling, glucocorticoid receptor signaling, mTOR signaling, PPARα/RXRα activation
	RARB	RAR activation, Wnt/β-catenin signaling
	SMO	Protein kinase A signaling, regulation of EMT pathway, Wnt/β-catenin signaling
	THBS1	Inhibition of TSP1, p53 signaling
	TNS1	FAK signaling
	TRPC5	Breast cancer regulation by Stathmin1
hsa-miR-551b	CASP2	Apoptosis, TNFR1 signaling
	ERBB4	ErbB signaling
	HES7	Notch signaling
	MEF2C	Calcium signaling, ERK5 signaling, p38 MAPK signaling, phospholipase C signaling, PPARα/RXRα activation
	NTRK2	NF-κB signaling, PTEN signaling

Targeted genes of miRNAs specific for the BRCA1-N to BRCA1-C transition are involved in several cellular processes associated with BRCA1 function, such as cell cycle regulation (BTG2, BTRC, CDK6, and E2F1), proliferation (ACTVR1B, BTRC, and DVL3), apoptosis (BCL2, FAS, TNF, and PTEN), but also less expected processes, including epithelial junctions and ECM interaction (ACTA2, ACVR1B, FGF1, PRKACB, and PTEN), cellular movement (FGF9 and PIK3R1), protein trafficking (DNM3, FGF1, FGF9, and RAB7A), and metabolism (FOXO3 and PTPN1).

Targeted genes of miRNAs specific for the BRCA2-N to BRCA2-C transition are involved in many similar processes, including epithelial junctions and ECM interaction (ACTA11, ACVR1B, CDLDN19, and FGF11), apoptosis (CASP2, and FAS), protein trafficking (FGF11, and ITGA2B), proliferation (CDH5, DACVR1B, DVL3, ERBB3, LRP6, and RPS20), cell cycle regulation (BTG2, CDK6, E2F2, PPP2R2A, and RBL2), and cellular movement (FGF11). Some of the targeted genes are shared with the BRCA1 axis (ACVR1B, BTG2, CDK6, DVL3, and FAS) or belong to the same family (e.g. FGFs or E2F), although they are regulated by different miRNAs. However, miRNAs deregulated in the BRCA2 axis show more targeted genes involved in estrogen receptor signaling compared to the BRCA1 axis. A more detailed overview of the targeted genes and associated pathways can be found in Supplementary table SIII.

### Comparison with published miRNA expression data

A systematic literature search yielded 1739 articles in PubMed. Six articles met the inclusion and exclusion criteria [22, 27, 30–33]. An overview of the selected studies is given in Table 11. Five studies used sporadic breast carcinomas, whereas the study of Tanic et al. (2012) [32] investigated miRNA expression in “familial” breast cancer, including patients with proven BRCA1 or BRCA2 germ-line mutations and non-BRCA1/2-associated familial carcinomas. Tumor-adjacent normal breast tissue, normal breast tissue from mammoplasty surgeries and normal breast tissue from prophylactic mastectomies were used as normal breast tissue controls in the different studies. Unfortunately, many patient characteristics could not be derived. The number of differentially expressed miRNAs was lower in all studies compared to the present study. Moreover, four out of six studies reported more up-regulated miRNAs compared to down-regulated miRNAs.

**Table 11:** Overview breast cancer miRNA expression profiling studies in literature

Author	Year	Journal	Country	N of samples	miRNA analysis						
					Platform	Total # miRNAs on array	GEO accession number	Cut-off criteria	# differentially expressed miRNAs	# up-regulated miRNAs	# down-regulated miRNAs
Chen	2013	PLoS One	USA	8 paired sporadic BC and pre-invasive/normal adjacent tissue; 16 unpaired sporadic BC	Human miRNA Microarray V3 (Agilent)	866	NA	*p* <= 0.05 (paired analysis); FDR <= 0.01 (unpaired analysis)	25	15	10
Iorio	2005	Cancer Res	Italy, USA	76 sporadic BC; 34 NBT	miRNA microarray V1.0 (KCI)	161	NA	FDR < 0.05	29	17	12
Ouyang	2014	PLoS One	China	3 triple negative BC; 3 adjacent NBT	miRCURY LNA Array 16.0	1513	NA	*p* <= 0.05	41	18	23
Tahiri	2014	Carcinogenesis	Norway	29 sporadic BC; 29 NBT	Human miRNA Microarray V3 (Agilent)	866	E-MTAB-779	FDR < 0.001	63	31	32
Tanic	2012	PLoS One	Spain	22 familial BC; 14 NBT*	miRCURY LNA microRNA Array Kit (Exiqon)	1276	GSE32922	FDR < 0.05	19	17	2
Yan	2008	RNA	China	8 paired sporadic BC normal adjacent tissue	CapitalBio	435	NA	FDR = 0; FC > 2.0	16	9	7

BC = breast cancer; NBT = normal breast tissue; NA = not available; FDR = false discovery rate. * 22 hereditary tumors (3 BRCA1, 5 BRCA2, 14 BRCAX) and 14 normal breast tissues (3 BRCA1, 5 BRCA2, and 1 BRCAX from prophylactic surgery; 5 normal from breast reductions).

The studies show little overlap in miRNA expression patterns: only 15 out of 92 (16.3%) up-regulated miRNAs and 15 out of 101 (14.9%) down-regulated miRNAs were reported by at least two studies (Table 12). Most frequently reported deregulated miRNAs were miR-21 (up-regulated in five studies), miR-155 (up-regulated in three studies), miR-145 (down-regulated in three studies) and miR-143 (down-regulated in three studies). Other up-regulated miRNAs, reported by two studies, were miR-181b, miR-98, miR-20a, miR-183, miR-141, miR-200b, miR-106b, miR-425, miR-149, miR-210, miR-1280, miR-29b and let-7f. Other down-regulated miRNAs, reported by two studies, were miR-205, miR-125b, miR-99a, miR-100, miR-195, miR-10b, miR-320c, miR-130a, miR-575, let-7d, miR-486-5p, miR-140-3p, and miR-335.

**Table 12:** Results miRNA expression data comparison from breast cancer miRNA expression profiling studies already available in literature

miRNA	Number of studies that consistently reported miRNA	Studies by author	Total number of samples	Average FC	Range FC
**Up-regulated miRNAs**					
hsa-miR-21	5	Chen, Iorio, Tahiri, Tanic, Yan	236	5.27	1.55-14.69
hsa-miR-155	3	Iorio, Tahiri, Yan	176	2.22	1.28-2.29
hsa-miR-210	2	Iorio, Tahiri	168	2.23	1.43-3.03
hsa-miR-149	2	Iorio, Tahiri	168	1.94	1.08-2.8
hsa-miR-183	2	Chen, Tahiri	82	6.50*	NA
hsa-miR-200b	2	Chen, Tahiri	82	4.18*	NA
hsa-miR-141	2	Chen, Tahiri	82	3.04*	NA
hsa-miR-425	2	Chen, Tahiri	82	2.31*	NA
hsa-miR-106b	2	Chen, Tahiri	82	2.07*	NA
hsa-miR-20a	2	Chen, Tahiri	82	1.68*	NA
hsa-miR-98	2	Tahiri, Yan	66	2.01	1.88-2.13
hsa-miR-181b	2	Tahiri, Yan	66	1.43*	NA
hsa-miR-1280	2	Ouyang, Tahiri	64	1.74*	NA
hsa-let-7f	2	Chen, Yan	32	2.39*	NA
hsa-miR-29b-3p	2	Chen, Yan	32	2.27*	NA
**Down-regulated miRNAs**					
hsa-miR-145	3	Iorio, Tahiri, Tanic	204	3.31	2.38-4.24
hsa-miR-143	3	Iorio, Tahiri, Tanic	204	1.48	1.10-1.85
hsa-let-7d	2	Iorio, Tahiri	168	1.53	1.12-1.94
hsa-miR-99a	2	Tahiri, Tanic	94	3.57*	NA
hsa-miR-125b	2	Tahiri, Tanic	94	3.42*	NA
hsa-miR-10b	2	Tahiri, Tanic	94	2.57*	NA
hsa-miR-100	2	Tahiri, Tanic	94	2.54*	NA
hsa-miR-205	2	Tahiri, Tanic	94	2.48*	NA
hsa-miR-195	2	Tahiri, Tanic	94	2.37*	NA
hsa-miR-130a	2	Tahiri, Tanic	94	2.01*	NA
hsa-miR-320c	2	Tahiri, Tanic	94	1.93*	NA
hsa-miR-575	2	Chen, Tahiri	82	2.26*	NA
hsa-miR-486-5p	2	Ouyang, Tahiri	64	4.86*	NA
hsa-miR-140-3p	2	Ouyang, Tahiri	64	3.15*	NA
hsa-miR-335	2	Tanic, Yan	44	2.43	1.40-3.45

* FC based upon a single value. NA = not applicable.

Marked in blue: miRNA also found differentially expressed in the same direction in BRCA1-C vs. BRCA1-N comparison in our analysis.

Marked in yellow: miRNA also found differentially expressed in the same direction in BRCA1-C vs. BRCA1-N and BRCA2-C vs. BRCA2-N comparisons in our analysis.

Marked in red: miRNA also found differentially expressed in the same direction in BRCA2-C vs. BRCA2-N comparison in our analysis.

Marked in green: miRNA also found differentially expressed in the opposite direction in BRCA1-C vs. BRCA1-N and BRCA2-C vs. BRCA2-N comparisons in our analysis.

Marked in purple: miRNA also found differentially expressed in the opposite direction in BRCA2-C vs. BRCA2-N comparison in our analysis.

Several of these miRNAs were also deregulated in our dataset (See Table 12, highlighted (colored) miRNAs), including some of the top 10 deregulated in miRNAs in BRCA1/2-associated breast carcinomas, as well as other miRNAs. Further explanations on the similarities and differences between miRNAs deregulated in sporadic and BRCA1/2-associated breast carcinomas will be made below.

## DISCUSSION

In the present study, miRNA expression profiles were investigated by miRNA microarray between normal and cancer tissue from BRCA1 and BRCA2 germ-line mutation carriers, in comparison with normal tissue from non-carriers. This yielded several biologically interesting findings. First, many more miRNAs were found to be differentially expressed between the carcinomas and asymptomatic normal breast tissue in BRCA1 and BRCA2 germ-line mutation carriers compared to the number of differentially expressed miRNAs between sporadic breast carcinomas and normal breast tissue as derived from the literature. This may be due to the extensive chromosomal instability seen in BRCA1/2-associated breast carcinomas, leading to loss of chromosomal regions and consequently, miRNA genes.

miRNAs deregulated in the BRCA1 and BRCA2 axes showed a similar chromosomal distribution, and several hotspots of down-regulated miRNAs were found in both axes. Indeed, about 21% of deregulated miRNAs matched reported locations of chromosomal instability in BRCA1/2-associated breast carcinomas and fragile sites in the genome [28]. The amount of miRNAs found at fragile sites in the genome was however lower than the >50% reported previously [29]. This could be explained by the fact that the chromosomal location of a considerable number of miRNAs from our analysis is still unclear. We found no clear indications whether miRNA deregulation in BRCA1/2-associated breast carcinomas could be due to direct effects of impaired BRCA1/2 function. For instance, pathway analysis did not yield specifically enriched pathways for the BRCA1 and BRCA2 axes. However, the BRCA2-C *vs*. BRCA2-N comparison yielded many more deregulated miRNAs compared to the BRCA1-C *vs*. BRCA1-N comparison. It was recently discovered that BRCA1 accelerates miRNA processing via interaction with Drosha [34]. Impaired BRCA1 function could therefore lead to less miRNA production.

We found several deregulated miRNAs in BRCA1*/2*-associated breast carcinomas that were also reported in studies investigating sporadic breast tumors, similar to the study by Tanic et al. (2012) [32]. This suggests the existence of miRNAs which are important in regulating oncogenes and tumor suppressor genes in both the hereditary and sporadic settings [32]. These miRNAs have been shown to play a role in cell proliferation and invasion, acting on HER signaling (miR-143, miR-145, miR-205) [35, 36], cell cycle regulation (miR-195) [37], epithelial to mesenchymal transition (miR-145, miR-205) [38, 39], and tumor angiogenesis (miR-145) [40]. Interestingly, several miRNAs that were up-regulated in sporadic breast carcinomas were also up-regulated in BRCA1-associated breast carcinomas from our analysis compared to normal breast tissues from BRCA1 germ-line mutation carriers. BRCA2-associated breast carcinomas did not show any similarities in up-regulated miRNAs with sporadic breast carcinomas, which is remarkable since BRCA2-associated carcinomas otherwise strongly resemble sporadic carcinomas. Mechanisms underlying these differences are currently unclear.

However, we also found many differentially expressed miRNAs between BRCA1/2-associated and sporadic carcinomas. This was mainly the case for up-regulated miRNAs, including miR-141 and miR-1280 (up-regulated in sporadic breast carcinomas and not deregulated in BRCA1/2-associated breast carcinomas); miR-1307-3p, miR-3162-3p, miR-155-5p, miR-4306, miR-185-5p, miR-574-5p, and miR-4455 (up-regulated in BRCA1-associated breast carcinomas compared to their normal counterparts, and not consequently deregulated in sporadic breast carcinomas); miR-4778-5p, miR-4433, miR-5010-5p, miR-1287, miR-663b, and miR-4688 (up-regulated in BRCA2-associated breast carcinomas compared to their normal counterparts, and not consequently deregulated in sporadic breast carcinomas); miR-3676-5p and miR-937-5p (up-regulated in BRCA1/2-associated breast carcinomas and not deregulated in sporadic breast carcinomas); and miR-320c and miR-486-5p (down-regulated in sporadic breast carcinomas and not deregulated in BRCA1/2-associated breast carcinomas). The function of most of these miRNAs in breast cancer is still unclear. However, miR-155 deregulation has been associated with drug resistance in breast cancer by repression of FOXO3a, stimulation of epithelial-to-mesenchymal transition and MAPK signaling [41]. Elevated miR-155 expression levels have been found in HER2-positive breast carcinomas [42]. Moreover, several miRNAs were deregulated in the opposite direction between BRCA1/2-associated breast carcinomas and sporadic breast carcinomas. These mostly entailed up-regulated miRNAs in sporadic breast carcinomas and down-regulated in BRCA2-associated breast carcinomas (miR-20a-5p, let-7f-5p, and miR-29b-3p). miR-20a-5p has been associated with triple negative tumors that showed enhanced expression compared to luminal A tumors [43]. miR-29b has been negatively associated with HER2 expression [44]. The function of let-7f is still unclear. Apart from potential underlying biological mechanisms, differences found in miRNA expression between BRCA1/2-associated and sporadic breast carcinomas are also to some extent likely to be due to technical differences (see below).

Second, several miRNAs were found to be already deregulated in the normal breast tissue from BRCA1 germline mutation carriers. Of these, miR-140-3p, miR-335, miR-320c and miR-486-5p have been previously described as deregulated in sporadic breast cancer. Recently, it has been reported that most miRNA deregulation occurs at a very early stage in sporadic breast carcinogenesis, during the transition from normal breast tissue to atypical ductal hyperplasia [27]. In case of an underlying BRCA1 or BRCA2 germline mutation, impaired DNA repair could already lead to aberrant miRNA expression in epithelial cells before these alterations lead to morphological changes. Additionally, miRNA deregulation at this stage could also be explained as the result of stromal changes. Deregulation of the microenvironment and intercellular interactions have an important role in breast neoplastic transformation [28]. It has been shown that the asymptomatic breast of BRCA1 and BRCA2 germ-line mutation carriers shows epithelial and stromal changes, including less differentiated lobules and a more dense and fibrotic intra-lobular stroma [28]. No miRNAs were deregulated in the normal breast tissue from BRCA2 germline mutation carriers compared to non-carriers. This could mean that miRNA deregulation along the BRCA2 axis occurs at a later stage compared to the BRCA1 axis. However, the BRCA2-C *vs*. Healthy-N comparison yielded 600 differentially expressed miRNAs and the BRCA2-C *vs*. BRCA2-N comparison ‘only’ 96 miRNAs. An explanation for the uneven distribution of differentially expressed miRNAs along the BRCA2 axis could be that subtle changes in miRNA deregulation could not be identified due to a small sample size.

Third, no significant correlations were found between miRNA expression profiles on the one hand and biological differences (such as age and ER status) on the other hand. This is in contrast with previously reported findings based on sporadic carcinomas [22, 24]. The miRNA changes caused by impaired BRCA1 or BRCA2 function might overrule the effect of these parameters on miRNA expression. However, we did not apply matching for sample selection and the sample size was relatively small. Therefore, a relation between age and ER status may have been missed.

Fourth, pathway analysis yielded several less expected processes in which deregulated miRNAs are predicted to be involved, such as epithelial junctions and ECM interaction, cellular movement, and protein trafficking. Dacheux *et al*. [45] recently discovered cellular movement and protein trafficking as possible new functions of BRCA1 as well. Weber *et al*. [28] showed extensive genomic instability in the cancer stroma in BRCA1/2-associated breast carcinomas. A genetically unstable stroma might facilitate neoplastic transformation in the breast epithelium. Moreover, it has recently been published that stromal components also show miRNA deregulation and are affected by miRNAs secreted from tumor cells [46–49]. However, the role of these less expected processes in BRCA1/2-associated breast carcinogenesis needs to be further examined.

Two miRNAs had a similar differential expression pattern in microarray and qRT-PCR analysis: miR-99a and let-7b. miR-99a was down-regulated in breast carcinomas compared to normal breast tissues in both BRCA1 and BRCA2 germ-line mutation carriers. miR-99a deregulation has been reported in several human cancers, including breast cancer, and is involved in apoptosis and epithelial-to-mesenchymal transition by regulation of the mTOR, Akt and TGF-β pathway [50–55]. TGF-β pathway activity is decreased by loss of miR-99a, resulting in increased proliferation and decreased migration [51]. Let-7b was found to be specifically down-regulated in invasive breast carcinomas of BRCA2 germ-line mutation carriers. Our target gene analysis showed that let-7b is involved in cellular pathways, including those of p38 MAPK, p53, Wnt/β-catenin, apoptosis, tight junction, integrin and actin cytoskeleton. A few studies have shown let-7b down-regulation in human cancers, including breast, gastric and lung cancer [56–59]. In breast cancer, low let-7b expression has been associated with poor prognosis [57], whereas high let-7b expression has been observed in luminal A tumors and is associated with a favorable prognosis [56]. Further research is needed to investigate whether let-7b might be able to distinguish BRCA2-associated from sporadic breast carcinomas. This would be very interesting as BRCA2-associated and sporadic breast carcinomas show many similarities in histology and protein expression.

There are some limitations inherent to the techniques used in this study. Whole tissue and not laser-captured micro-dissected tissue samples have been used for miRNA profiling. This could have influenced the results, as miRNA profiling on whole tissue samples reflect changes in both epithelial and stromal cells [60]. However, we used maximally enriched samples by scraping off specific relevant regions from sections marked by a pathologist, so we do not believe that this has played a major role. Further, microarray technology does not show a distinct boundary between up-regulated and down-regulated miRNAs, but rather shows a broad distribution of miRNA levels. Until now, it is unclear which level is biologically functional for each miRNA. Lastly, the complex nature of microarray data makes the analysis highly dependent on bio-informatics and statistics [24]. This could, together with other factors such as the use of different microarray platforms, explain the little overlap between the findings of different miRNA profiling studies as shown in the systematic literature analysis [24].

In conclusion, this study revealed multiple deregulated miRNAs in BRCA1/2-associated breast carcinomas, some shared with sporadic breast carcinomas, but several possibly specific to BRCA1/2 carcinogenesis. Specific deregulated miRNAs in BRCA1/2-associated carcinomas appear to target similar pathways. This suggests the existence of common targetable miRNA regulated pathways driving BRCA1/2-associated breast carcinogenesis. These findings warrant further studies on the role of these miRNAs in BRCA1/2-associated breast carcinogenesis.

## MATERIALS AND METHODS

### Patient samples

For miRNA microarray analysis and initial qRT-PCR validation, a total of 46 FFPE patient samples were obtained from the archives of the University Medical Center Utrecht (UMCU), University Medical Center Groningen, the Familial Cancer Clinic of the VU University Medical center Amsterdam, and of local hospitals around Utrecht. Since we used archival pathology material which does not interfere with patient care and does not involve the physical involvement of the patient, no ethical approval is required according to Dutch legislation: the Medical Research Involving Human Subjects Act (Wet Medisch Wetenschappelijk Onderzoek met Mensen) [61]. Use of anonymous or coded left over material for scientific purposes is part of the standard treatment contract with patients and therefore informed consent procedure was not required according to our institutional medical ethical review board. This has also been described by van Diest et al. [62]. The patient samples have already been described under results and are shown in Tables 1a and 1b.

### RNA isolation

Whole tissue sections (10 μm thick) were cut from the paraffin tissue blocks for RNA isolation. Total RNA was extracted using the miRNeasy FFPE Kit (Qiagen, Venlo, The Netherlands) according to the manufacturer's protocol. For the tumor samples, the tumor area in five sections was scraped off from the slides, guided by haematoxylin and eosin stainings of the tissue samples. For the normal breast tissue samples, the whole area of the 10 tissue sections was scraped off. RNA concentration and integrity were determined using the 2100 Bioanalyzer system (Agilent Technologies, Santa Clara, CA, USA).

### miRNA microarray analysis

### miRNA microarray technique

miRNA microarray analysis was performed at the Sidney Kimmel Comprehensive Cancer Center Microarray Core Facility at the Johns Hopkins University, Baltimore, USA. The Human miRNA Microarray v19.0 (Agilent Technologies), containing 2006 human miRNAs from the miRBase database (release 19.0), was used. Sample preparation and hybridization were done according to manufacturer's instructions (for details, see Supplementary methods). Raw data were processed and analyzed using GeneSpring GX v12.5 (Agilent Technologies). Median fluorescence intensity values smaller than a threshold of 1 were set equal to 1. Probe level data were log2 transformed and normalized to the 75^th^ percentile based upon the distribution of signal intensities. miRNA probes were included if 100 percent of the samples in any 1 out of the 5 classes had normalized expression values within the 50th and 100th percentile in order to remove miRNAs showing low expression and little variation between the samples. To correlate miRNA expression with BRCA status or other clinicopathologic characteristics, the unpaired *t*-test for unequal variance or ANOVA for unequal variance were used, depending on the number of groups to compare. In order to correct for multiple comparisons, adjusted *p*-values were obtained by using Benjamini and Hochberg's FDR. Level of significance was set at FDR < 0.05.

### Chromosomal distribution analysis

The chromosomal distribution of differentially expressed miRNAs between classes was analyzed using Fisher's exact test, performed in SPSS v20 (IBM, Armonk, NY, USA). Only miRNAs of which the localization in the genome was known were included in this analysis.

### qRT-PCR

All patient samples were diluted to a total RNA concentration of 2 ng/μl. For reverse transcription of specific miRNAs, the TaqMan microRNA Reverse Transcription kit together with microRNA Assays was used according to the manufacturer's protocol (Applied Biosystems, Life Technologies, Carlsbad, CA, USA). The following thermal cycler conditions were used for reverse transcription: 30 min at 16°C, 30 min at 42°C, 5 min at 85°C, followed by 4°C. miRNA expression was investigated using the TaqMan Universal PCR Master Mix II without UNG together with microRNA assays (Applied Biosystems). The following thermal cycler conditions were used for qRT-PCR: 10 min at 95°C, followed by 40 cycles (15 s at 95°C, and 60 s at 60°C). The microRNA Assays (Applied Biosystems) consisted of the following primer sets (5X primer for reverse transcription, 20X primer for qRT-PCR): hsa-miR-99a-3p, hsa-miR-210, hsa-miR-21-5p, hsa-miR-183-5p, hsa-miR-378a-5p, hsa-miR-153, hsa-miR-4778-5p, hsa-miR-4443, hsa-let-7b-5p, hsa-miR-551b-3p. All samples were tested in duplicate. Reverse transcription and qRT-PCR for samples of the initial cohort were done on the IQ5 Multicolor Real-Time PCR Detection System and C1000 Touch thermal cycler with CFX96 Real-Time system (both Bio-Rad, Hercules, CA, USA), respectively. For the second cohort, the Veriti 96 Well Thermal Cycler (Applied Biosystems) and the ViiA™ 7 Real Time PCR System (Life Technologies) were used for reverse transcription and qRT-PCR, respectively. The qRT-PCR data of the first cohort were normalized to the second cohort by the use of replicates. Expression levels were calculated based on the comparative threshold cycle (ΔΔ Ct) method and were normalized to miR-125a-5p. The statistical analysis, *i.e*. the Kruskal-Wallis test to compare the median of the normalized Ct values between the classes, was performed in SPSS v20 (IBM). Level of significance was set at p ≤ 0.01, due to multiple comparisons.

### Cluster analysis

Clustering was carried out on both samples and miRNAs using a Self-Organizing Map in Cluster 3.0 (Eisen Lab, University of California, Berkeley) using all samples and all miRNAs that passed the filtering criteria. Default settings were used: a grid of 6×6, 100.000 iterations, and an initial learning rate of 0.02 for clustering genes; a grid of 3×3, 20.000 iterations, and an initial learning rate of 0.02 for clustering samples. The Self-Organizing Map was carried out using the Pearson centered distance algorithm with complete linkage rule. For visualization of the dendrogram, Java Treeview (http://jtreeview.sourceforge.net/) was used.

### Target gene and pathway analysis

Target gene and pathway analysis were performed using the web-based computational tool DIANA-mirPath v2.0 (http://www.microrna.gr/miRPathv2) and Ingenuity Pathway Analysis (IPA) (Ingenuity Systems, http://www.ingenuity.com/products/ipa). DIANA-mirPath was used to investigate the combinatorial effect of multiple miRNAs on pathways. Differentially expressed miRNAs between invasive breast carcinomas and normal breast tissues from prophylactic mastectomies for both BRCA1 and BRCA2 germ-line mutation carriers with FC ≥ │2.0│and FDR < 0.05 (unpaired *t-*test for unequal variance) were selected for this analysis. The Pathways Union mode was used with micro-T-threshold of 0.8 and level of significance was set at *p* < 0.05 with FDR correction applied. miRNAs selected for qRT-PCR validation were subjected to IPA, which combines the results from several prediction tools, including TarBase, miRecords, and TargetScan. Only experimentally validated and highly predicted targets were considered. Level of significance of the Fisher's exact test was set at *p* < 0.05.

### Comparison with miRNA expression data available in literature

**Figure 4:**
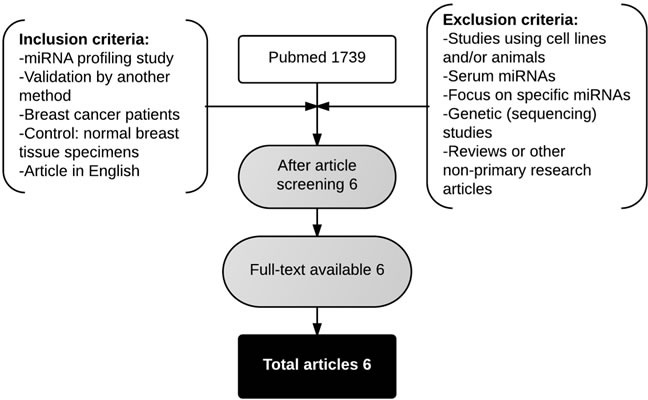
Flowchart showing the article selection strategy to attain breast miRNA expression profiling studies

Breast cancer miRNA expression profiling studies were identified using the PubMed database. The search syntax is shown in Table 13. Inclusion and exclusion criteria were used to screen the articles and are mentioned in Figure 4. The following data were extracted from the studies: author, year of publication, journal, location of study, selection and characteristics of patient samples, miRNA microarray platform, cut-off criteria used for statistical analysis, and lists of up- and down-regulated miRNAs including p-value and fold change (if available). The differentially expressed miRNAs reported by the studies were ranked according to the following protocol [63–65]: (1) number of studies that reported the miRNA and with a consistent direction of expression change; (2) total number of samples from studies in agreement upon which the differential expression of the miRNA is based; (3) average fold change from studies in agreement. For the ranking, an online bio-informatics tool for comparing lists (http://bioinfogp.cnb.csic.es/tools/venny/) was used.

**Table 13:** Overview search strategy for breast cancer miRNA expression profiling studies

Database	Search syntax
PubMed Date: Nov 23, 2014	((((((((mirna[Title/Abstract]) OR mirnas[Title/Abstract]) OR microrna[Title/Abstract]) OR micrornas[Title/Abstract]) OR mir[Title/Abstract]) OR mirs[Title/Abstract])) AND (breast[Title/Abstract])) AND ((((((cancer[Title/Abstract]) OR cancers[Title/Abstract]) OR tumor[Title/Abstract]) OR tumors[Title/Abstract]) OR tumour[Title/Abstract]) OR tumours[Title/Abstract])

## SUPPLEMENTARY MATERIAL TABLES





## Abbreviations

**General**
BRCA1/2: BRCA1 and BRCA2
ECM: Extra-cellular matrix
ER: Estrogen receptor
FC: Fold change
FDR: False Discovery Rate
FFPE: Formalin-fixed paraffin-embedded
IPA: Ingenuity Pathway Analysis
PR: Progesterone receptor

## Classes

BRCA1-C-BRCA1-associated invasive breast carcinomas

BRCA2-C-BRCA2-associated invasive breast carcinomas

BRCA1-N-Normal breast tissue from BRCA1 germ-line mutation carriers

BRCA2-N-Normal breast tissue from BRCA2 germ-line mutation carriers

Healthy-N-Normal breast tissue from non-mutation carriers
