# Supplementary material for: miRNA expression patterns in normal breast tissue and invasive breast cancers of BRCA1 and BRCA2 germ-line mutation carriers
Source: Oncotarget. 2015 Sep 11;6(31):32115–37. doi: 10.18632/oncotarget.5617 (PMC4741663; doi:10.18632/oncotarget.5617)
Supplement: Supplementary file 1 [file oncotarget-06-32115-s001.pdf]

miRNA expression patterns in normal breast tissue and invasive breast cancers of BRCA1 and BRCA2 germ-line mutation carriers

Supplementary Material

OVERVIEW SUPPLEMENTARY DATA

Methods.	Characterization of patient samples and details of miRNA microarray analysis
Table SI.	Differentially expressed miRNAs between HER2 positive and negative breast carcinomas
Table SII.	Pathway analysis - detailed DIANA-mirPath results (see separate file)
Table SIII.	Target gene analysis - detailed IPA results (see separate file)

The following files can be supplied upon request:

1. Raw data files miRNA microarray (non-normalized)

2. Processed data files miRNA microarray (normalized) that formed the basis of this research

SUPPLEMENTARY METHODS

Characterization of patient samples

From the paraffin tissue blocks, sections (4 µm thick) were cut and stained with haematoxylin and eosin for histopathology. Immunohistochemistry was performed to determine the ER, PR and HER2 status as part of routine pathology to guide clinical decisions concerning adjuvant therapy. For ER, a rabbit monoclonal anti-human antibody was used (1:100, pre-treatment with EDTA buffer) (DAKO, Cambridge, UK). For PR, a rabbit polyclonal anti-human antibody was used (1:100, pre-treatment with citrate buffer) (DAKO). For HER2, a mouse monoclonal anti-human antibody was used (1:100, pre-treatment with EDTA buffer) (Neomarkers, Kalamazoo, Massachusetts, USA). The slides were reviewed and scored by a single experienced breast pathologist (PJvD).

Details of miRNA microarray analysis

At first, RNA analysis was performed to determine total RNA and miRNA concentrations and the 28S and 18S ribosomal RNA bands, using the 2100 Bioanalyzer and software (Agilent, Santa Clara, CA, USA). 150 ng of total RNA was dephosphorylated using 11.2 units of calf intestine alkaline phosphatase (30 min at 37°C), followed by end-labeling with pCp-Cy3 and 15 units of T4 RNA ligase (2 hours at 16°C), using the miRNA Complete Labeling and Hyb Kit (Agilent Technologies). Labeled samples were then purified using Micro Bio-Spin 6 columns (Bio-Rad, Hercules, CA, USA). Measurements of labeling efficiency and nucleic acid concentration were performed using Nanodrop ND-1000 spectrophotometer. 10x blocking agent and 2x Hi-RPM hybridization buffer were added to the samples (Agilent Technologies). Hybridization was carried out for 20 hours at 55°C with rotation at 20 rpm in a designated Agilent G2545A hybridization

oven. Afterwards, microarrays were washed and scanned using an Agilent scanner with Agilent Scan Control 7.0 software. Probe level data were extracted by the Agilent Feature Extraction Software v9.5.3.1 for miRNA microarray (Agilent Technologies). For each array, a Feature Extraction file (containing signal intensities from all individual probes), a GeneView file (containing summarized signal intensities for each miRNA by combining replicate probe intensities), and a quality control report were generated. Feature extraction files were imported into GeneSpring GX v12.5 software (Agilent Technologies) for analysis.

Table SI. Differentially expressed miRNAs between HER2 positive and negative breast carcinomas		
**miRNA**	**FDR**	**FC**
**Up-regulated**		
hsa-miR-1225-3p	0.002578	1.356661
hsa-miR-1225-5p	0.049636	1.252916
hsa-miR-1234-5p	0.047666	1.233338
hsa-miR-1268b	0.049636	1.288895
hsa-miR-1273f	0.011607	1.276324
hsa-miR-1275	0.033476	1.192103
hsa-miR-134	0.016774	1.390645
hsa-miR-150-3p	0.002116	1.296638
hsa-miR-1587	0.016009	1.448497
hsa-miR-187-5p	0.005653	1.435179
hsa-miR-2392	0.047346	1.351058
hsa-miR-3141	0.016009	1.187987
hsa-miR-345-3p	0.047346	1.43803
hsa-miR-3659	0.032904	1.224634
hsa-miR-371a-5p	0.026413	1.385174
hsa-miR-3937	0.035898	1.306349
hsa-miR-3960	0.043308	1.313168
hsa-miR-4253	0.009594	1.34886
hsa-miR-4314	0.032904	1.253843
hsa-miR-4327	0.039993	1.40093
hsa-miR-4417	0.004928	1.42873
hsa-miR-4463	0.016009	1.306708
hsa-miR-4486	0.002116	1.464665
hsa-miR-4534	0.041282	1.221951
hsa-miR-4655-5p	0.005653	1.315056
hsa-miR-4689	0.001457	1.457825
hsa-miR-4701-3p	0.015116	1.459531
hsa-miR-4738-3p	0.016009	1.288022
hsa-miR-4758-5p	0.03821	1.357123
hsa-miR-5003-3p	0.049636	1.459127
hsa-miR-5090	0.002116	1.214843
hsa-miR-5787	0.007037	1.325038
hsa-miR-601	0.032904	1.321849
hsa-miR-6089	0.018909	1.219043
hsa-miR-6511a-5p	0.015116	1.317875
hsa-miR-6511b-5p	0.026413	1.368215
hsa-miR-671-5p	0.033476	1.397388
hsa-miR-6722-3p	0.012103	1.288777
hsa-miR-718	0.016009	1.365627
**Down-regulated**		
hsa-let-7a-3p	1.07E-04	8.70545
hsa-let-7a-5p	0.005648	2.332647
hsa-let-7b-5p	3.94E-04	2.588086
hsa-let-7d-5p	0.047346	1.852211
hsa-let-7e-5p	0.048299	2.50663
hsa-let-7f-5p	0.004928	2.336887
hsa-let-7g-5p	0.016009	2.059855
hsa-miR-1248	0.004902	5.868749
hsa-miR-125a-5p	0.021969	2.47606
hsa-miR-152	0.026413	2.517945
hsa-miR-193a-3p	2.31E-04	2.850227
hsa-miR-214-5p	0.016009	6.755226
hsa-miR-219-5p	0.016009	27.23514
hsa-miR-22-3p	0.017375	2.013559
hsa-miR-22-5p	0.016774	5.475135
hsa-miR-26b-5p	0.032904	3.013432
hsa-miR-27b-3p	0.033476	1.930875

hsa-miR-29b-3p	0.035898	2.955335
hsa-miR-30b-5p	0.021969	2.979495
hsa-miR-30c-5p	0.047346	2.238031
hsa-miR-30d-3p	0.005653	13.89108
hsa-miR-30e-5p	0.036069	1.78208
hsa-miR-32-5p	0.002578	8.166767
hsa-miR-326	0.002369	6.961307
hsa-miR-335-3p	0.047346	26.41262
hsa-miR-339-5p	0.016009	4.567096
hsa-miR-33a-5p	0.032904	4.122062
hsa-miR-340-3p	0.019699	27.00444
hsa-miR-34a-3p	0.002116	12.77391
hsa-miR-34c-5p	2.31E-04	12.46885
hsa-miR-361-5p	0.01049	1.676426
hsa-miR-365a-3p	2.31E-04	3.368948
hsa-miR-374c-5p	0.007886	6.656583
hsa-miR-378h	0.02614	5.675561
hsa-miR-3944-5p	0.041476	3.142477
hsa-miR-409-3p	0.033168	4.38263
hsa-miR-450a-5p	0.007037	8.187235
hsa-miR-491-3p	0.010678	4.368222
hsa-miR-502-5p	0.016774	5.166341
hsa-miR-513c-3p	0.047346	3.762527
hsa-miR-5585-5p	0.016009	4.402581
hsa-miR-598	0.016774	36.40213
hsa-miR-652-3p	0.041282	1.980695
hsa-miR-744-5p	0.012103	5.111528
hsa-miR-95	0.007886	10.01408
hsa-miR-98-5p	0.014883	2.228227
